# Differences in Determinants Influencing Self-Rated Oral Health in Korean and American Adults

**DOI:** 10.3390/ijerph19063618

**Published:** 2022-03-18

**Authors:** Jeong-Ah Choi, So-Jung Mun, Won-Gyun Chung, Sun-Young Han

**Affiliations:** 1 Department of Health Administration, Graduate School, Yonsei University, 1 Yonseidae-gil, Wonju 26493, Gangwon-do, Korea; wjddkqhfk@yonsei.ac.kr; 2 Department of Dental Hygiene, Graduate School, Yonsei University, 1 Yonseidae-gil, Wonju 26493, Gangwon-do, Korea; 3 Department of Dental Hygiene, College of Software and Digital Healthcare Convergence, Yonsei University, Wonju 26493, Gangwon-do, Korea; sojung77@yonsei.ac.kr (S.-J.M.); wgchung@yonsei.ac.kr (W.-G.C.)

**Keywords:** self-rated oral health, objective oral health status, KNHANES, NHANES

## Abstract

This study aims to identify the differences in the determinants that influence self-rated oral health (SROH) among Korean and American adults aged 20 years or older and the differences in objective oral health status between Korea and the United States. It included 13,068 Koreans and 5569 Americans who participated in the seventh Korea National Health and Nutrition Examination Survey and the 2017–2018 National Health and Nutrition Examination Survey. All analyses were conducted using the SPSS 25 program. The 39% of Koreans and 27.7% of Americans rated their oral health as “poor”. The mean SROH score was lower in Korea (2.66) than in the US (3.15). Conversely, objective oral health was better among Koreans. Further, an analysis of the differences in the predictors of SROH between the two countries confirmed that there were significant differences in age, household income, education level, insurance type (none), type of smoking, self-rated health, and decayed teeth index. Government-led projects or policy-based changes that can improve objective oral health status are needed to boost SROH in Korea, and subsequent studies should examine other objective oral health indices (e.g., periodontal disease) as well as differences in sociocultural backgrounds between countries.

## 1. Introduction

Similar to self-rated health (SRH) [1], which is an index of various aspects of an individual’s health status, self-rated oral health (SROH) is a comprehensive index of one’s oral health status. SROH is an index with high reliability and validity for objective oral health status (e.g., periodontal disease, lost teeth) comparable to the results of an oral examination performed by a dentist [2,3,4]. SROH reflects the oral health status of an individual, the person who can best recognize the oral status from the onset of the disease until receiving treatment from the dentist [5,6]; thus, SROH may be a potential indicator for predicting oral diseases [7]. Therefore, SROH is very important because it is a proxy variable for actual oral health condition, and it can be used as an indicator for predicting oral diseases.

Previous studies have reported a gap in SRH status between Korea and the US [8,9]. According to the Organization for Economic Cooperation and Development Statistics, Korea has poorer SRH but better objective health status (prevalence of chronic disease, incidence of infectious disease) compared to the US [10,11]. Such differences in SRH and disease incidences between countries raise suspicion about the method by which the citizens of these countries rate their own health status [9].

A similar trend to SRH appears in SROH in Korea and US. A 2015 report by the American Dental Association indicated that 70% of American adults self-rated their oral health to be “very good/good”. In contrast, only 44.5% of Korean adults participating in the Korea National Health and Nutrition Examination Survey (KNHANES) for the same year self-rated their oral health to be “excellent/good,” indicating that Korean adults generally had average or poor SROH [12]. This can be related to the international gap in SRH status. As previously mentioned, the SROH of an individual is influenced by a variety of factors, including demographic, socioeconomic, and behavioral factors, as well as objective oral health and oral health awareness. In addition, SROH may differ among countries because of the ethnic and cultural backgrounds unique to each country and different health insurance systems [5]. Studies that compare differences between countries have the advantage of examining problems from more diverse perspectives than those that only analyze one country’s population [9], which allows for exploration of the factors affecting the SROH of Korean compared to foreign countries and to use them for policy judgment for development. However, most existing studies have been on the influencing factors of SROH in a single country, while no comparative analyses have been conducted on the various influencing factors between countries [5,6,13,14,15]. Meanwhile, studies have examined the differences in the influencing factors between countries with respect to SRH, which is a similar indicator [8,9].

Therefore, this study aimed to examine the determinants of SROH in adults in Korea and the US using the same parameters shown in the Korea National Health and Nutrition Examination Survey (KNHANES) and the National Health and Nutrition Examination Survey (NHANES), to identify the features of factors that influence SROH in each country and examine whether the gap in SROH between the two countries is actually related to objective oral health.

## 2. Materials and Methods

**Figure 1:**
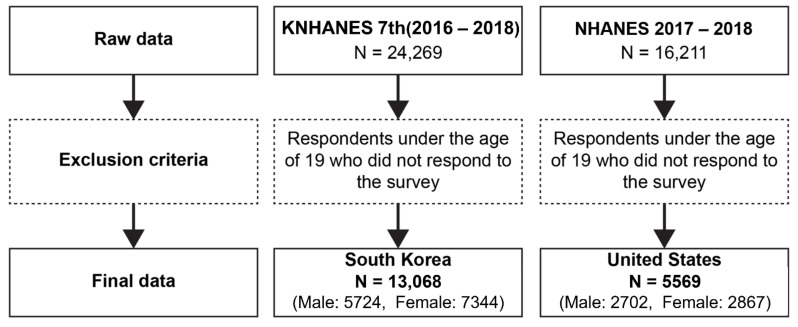
Process of selecting subjects.

This study included data from the KNHANES Ⅶ (2016–2018) of 13,068 Korean adults aged 20 years or older and from the NHANES 2017–2018 of 5569 American adults aged 20 years or older who participated in both the survey and health examination (Figure 1) [8,9]. The data used were from the latest available chronological match between the two countries.

### 2.1. Source of Data

KNHANES Ⅶ (2016–2018) is a national survey conducted in accordance with Article 16 of the National Health Promotion Act. Since its launch in 1998, approximately 10,000 people have been surveyed every year, and 24,269 individuals in 10,611 households participated in the KNHANES VII. The KNHANES was investigated for three years and the seventh dataset was completed. Per the KNHANES, a health survey, examination, and nutrition survey were performed on Korean nationals aged 1 year or older who reside in the Republic of Korea. The health survey and oral examination in this survey were performed at a mobile examination center, and the nutrition survey was conducted by visiting the target households in person. The stratified two-stage cluster sampling method was used, with survey tools and primary sampling unit (PSU) as the first extraction unit, and households as the second extraction unit. In the case of the KNHANES Ⅶ (2016–2018), the extraction frame was stratified based on the type of house. The weight of the health survey and examination survey (itvex), and the weight of the oral examination (oe) were used for analysis [16].

The 2017–2018 NHANES, a national survey, was conducted by the National Center for Health Statistics (NCHS) at the Centers for Disease Control and Prevention (CDC). Since its launch in the early 1960s, approximately 5000 people have been surveyed over two years. In 2017–2018, 16,211 people were selected as potential candidates, 9254 of whom completed the interview survey and 8704 of whom completed the examination. The NHANES consists of a nutrition survey and a health-related survey conducted at home and in a mobile examination center (MEC). The stratified two-stage cluster sampling method was used, with survey tools and PSU as the first extraction unit, and secondary sampling unit (SSU) as second extraction unit [17].

### 2.2. Independent Variables

To identify the determinants that influence SROH among Korean and American adults and to identify the differences in objective oral health status between the two countries, variables associated with socio-demographics, general and oral health, and SROH were selected.

#### 2.2.1. Sociodemographic Factors

Based on previous studies, gender, age, household income, education level, economic activity, and insurance type were selected as sociodemographic factors [8,9].

Household income was divided into high, medium-high, medium-low, and low based on the household income quartiles in Korea [16] and the family poverty income ratio (PIR) in the US. US household income was divided into low (PIR < 1.00), medium-low (1.00 ≤ PIR < 2.00), medium-high (2.00 ≤ PIR < 3.00), and high (PIR ≥ 3.00) categories [18,19].

Education level was divided into “≤middle school”, “high school”, and “≥university or college” according to the educational levels in Korea. Individuals who completed the curriculum for up to the third year of high school in Korea and grades 1–12 in the US were identified as the “high school” group. For both Korea and the US, economic activity was based on whether the individual was currently employed or not.

For insurance type, subscribers of employment and regional insurance were considered to have health insurance in Korea, and subscribers of private or Medicare were considered to have health insurance in the US. Medical aid recipients in Korea and Medicaid recipients in the US were classified as the “Medicaid” group. Individuals with no health insurance in Korea and the US were classified in the “None” group.

#### 2.2.2. General Health Factors

General health factors included chronic disease status, SRH, and type of smokers.

For chronic disease status, individuals having any one of the 13 chronic diseases used in a previous study (hepatitis B/C, thyroid disease, hypertension, arthritis, stroke, diabetes mellitus, myocardial infarction, hyperlipidemia, chronic obstructive pulmonary disease, asthma, angina, and cancer) [9] were classified in the “Yes” group. For cancer, gastric cancer, colorectal cancer, lung cancer, liver cancer, and thyroid cancer were included, after excluding other types of cancer with differences between the genders [20].

SRH was divided into “good” and “poor”, and type of smokers was divided into “Current smoker”, “Former smoker”, and “Never smoked” groups.

#### 2.2.3. Oral Health Factors

Oral health factors included the number of present teeth; decayed, missing, filled teeth (DMFT) index; decayed teeth (DT) index; filled teeth (FT) index; and missing teeth (MT) index, which were calculated based on 28 permanent teeth excluding third molars.

In Korea, dentition status was examined through an oral examination. Among the six tooth surfaces (buccal, distal, occlusal, mesial, and lingual/palatal) of each tooth, sound surfaces, decayed surfaces, filled surfaces, sealant surfaces were classified when the corresponding tooth currently exists. In the United States, data responding to “permanent tooth present” in the item surveyed on the number of teeth through oral examination were classified as Present Teeth (PT). When Root Rest was investigated, it was classified as no PT.

The DMFT index is the sum of individual DMFT (sum of the number of decayed, missing due to caries, and filled teeth in the permanent teeth) values divided by the sum of the population [21]. The DT index is the sum of individual DT (the number of decayed teeth) value divided by the sum of the population. The MT index is the sum of individual MT (the number of missing teeth due to caries) value divided by the sum of the population. The FT index is the sum of the individual FT (the number of filled teeth) value divided by the sum of the population. Teeth having both caries lesions (surfaces with ‘D’ component) and restorations/fillings (surfaces with ‘F’ component) were classified as DT in both countries.

### 2.3. Dependent Variables

#### Self-Rated Oral Health (SROH)

SROH was rated using a five-point Likert scale in both Korea (excellent, good, fair, poor, very poor) and the US (excellent, very good, good, fair, poor). In this study, the parameter was categorized to a binary variable to be used as an outcome variable in logistic regression. According to prior research, “fair” in Korea connotes a weak but positive meaning, whereas “fair” in the US has a negative connotation [9]. Hence, based on prior research, “Overall, how would you rate the health of your teeth and gums?” were categorized into excellent, good and fair into “good”, and poor and very poor into “poor” for Korea; and excellent, very good and good into “good”, and fair and poor into “poor” for the US [8,9].

### 2.4. Statistical Analysis

All analyses were performed using the SPSS 25.0 program (IBM SPSS Statistics, IBM, Chicago, IL, USA). Complex sample chi-square analysis was performed to examine the differences in SROH according to sociodemographic factors and general health factors between Korean and US adult populations. The differences in the means of oral health factors (number of present teeth, DMFT index, DT index, MT index, FT index) according to SROH in the two populations were analyzed using complex sample descriptive statistics. Lastly, the effects of general factors and health-related factors (general and oral health) on SROH were analyzed using complex sample logistic regression. Statistical significance was set at *p* ≤ 0.05.

The data used in this study were secondary data surveyed by the KDCA (Korea) and CDC (US) and can be accessed by the general public. Therefore, this study was conducted after receiving IRB review exemption from the Institutional Review Board of Wonju Severance Christian Hospital, Yonsei University, Korea on 25 February 2020 (approval number: CR319362).

## 3. Results

**Figure 2:**
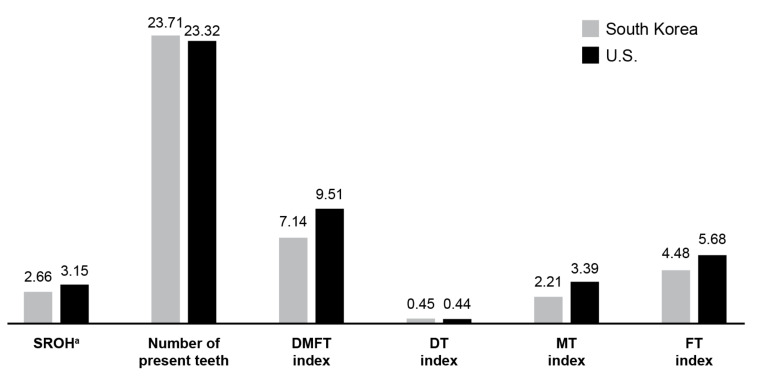
Oral health factors in Korean and American adults. ^a^: Self-rated oral health (SROH) was surveyed using a 5-point Likert scale.

Complex sample descriptive analysis revealed that 61.2% of Korean and 72.3% of US adults rated their oral health as “good”, while 38.8% of Korean and 27.7% of US adults rated their oral health as “poor”. Additionally, the mean SROH score (5-point Likert scale) was lower in Korea (2.66) compared to the US (3.15) (Table 1 and Figure 2).

Overall, it can be seen that Korean adults rated their oral health as poorer than their US counterparts. However, in the comparison of oral health factors between the two countries, objective oral health status (number of present teeth, DMFT index, MT index, FT index) was better in Korea than in the US (Figure 2). In terms of the differences in SROH based on oral health factors, all oral health factors for both the “good” and “poor” SROH groups were better in Korea than in the US. Further, the differences in the mean objective oral health factors between the “good” and “poor” SROH groups were greater in Korea than in the US (Table 2).

The effects of general characteristics (sociodemographic factors) and health factors (general health and oral health factors) on SROH were analyzed using complex sample logistic regression. Gender, economic activity, insurance type, chronic diseases, number of present teeth, MT index, and FT index were not significantly associated with SROH. In an analysis of the differences in the predictors of SROH between the two countries, there were significant differences in age, household income, education level, insurance type, type of smokers, SRH and DT index. The odds ratio (OR) increased with age, but decreased starting from the age of 70 years and older in Korea; however, no such continuous results were found in the US. In both Korea and the US, OR increased with lower household income, but it was much higher in the US (OR = 2.54, *p* < 0.001) than in Korea (OR = 1.49, *p* < 0.001). When SRH was poor, the OR for poorer SROH was 1.79 (*p* < 0.001) in Korea and 3.32 (*p* < 0.001) in the US, the latter showing a greater OR than that of the former. Among the oral health factors, the factor with the greatest influence was the DT index, with an OR of 1.47 (*p* < 0.001) in Korea and that of 1.69 (*p* < 0.001) in the US, also showing a higher risk in the US. The strongest predictor of SROH was age, followed by type of smokers, SRH, household income, and DT index in Korea, and SRH, followed by household income, type of smoking, age, and education level in the US (Table 3).

## 4. Discussion

This comparative study used data from KNHANES Ⅶ (2016–2018) and NHANES (2017–2018) to identify the determinants that influence SROH and the differences in objective health status between Korea and the US. To achieve the purpose of this study, the comparable country selected was the United States, a country that has a similar data set to that of Korea, but which has a difference in SROH.

The average score of SROH between countries was 2.66 for Korea and 3.15 for the US, confirming that Korea evaluated more negatively. However, the objective oral health factors were better in Korea than in the US. These results imply that Koreans evaluate SROH more negatively, even though the objective oral health status is better than that of the US. In addition, there were significant differences in age, household income, education level, insurance type, type of smokers, SRH, and DT index as predictors of SROH between the two countries. The OR for negative SROH consistently increased with age in Korea, whereas the same did not hold true for the US. Moreover, the OR for negative SROH decreased starting from the age of 70 years in Korea and 60 years in the US, but a previous study reported that elderly people with poor oral health have a general tendency to overrate their oral health status [22]. Thus, to promote proper oral health management according to age and improve SROH, it is important to develop policies pertaining to the prevention and management of oral diseases, implement various oral health education programs, and establish systems to promote continuous social involvement [23,24].

Both countries showed a consistent trend of SROH in terms of education level and household income. These results reflect the sociocultural characteristics of both countries, in which the education level translates to household income level. Moreover, individuals with lower education levels and household income tend to have negative SROH [5,25,26], while the difference in SROH according to household income becomes more severe with increasing income [26]. Considering that OR was higher in the US than Korea, there seems to be a difference in the social security systems between the two countries. In the US, many aspects related to social security depend heavily on the market, with relatively little governmental involvement [27]. Medicare does not cover most types of dental care [28] and there are predetermined amounts for each type of dental care. Therefore, most people receive dental coverage from private insurance. However, even private dental insurance has a high out-of-pocket cost, and dental coverage does not include employer-sponsored health insurance. As a result, many people choose not to seek dental care [27].

Conversely, Korea has a medical aid system for the low-income class, which covers the entire cost of care minus the legal out-of-pocket cost for the recipients stipulated by the law [29], which would increase the accessibility to health care compared to the US. According to OECD Health Statistics (2021), the number of doctors consultations per capita is 4.0 in the US. With 17.2 per capita, Korea is the largest among OECD countries, far exceeding the OECD average of 6.8. In addition, the number of dentists consultations per capita was 1.1 in the US and 1.6 in Korea (OECD average of 1.2), with Korea having more consultations for all medical and dental clinics [10]. Therefore, such differences in the social security systems between the two countries may have contributed to Korea having a lower OR for negative SROH than the US, despite having a lower education level and household income.

The results of insurance types can also be seen as differences in the social security systems between countries. In Korea, the entire nation receives medical insurance through the National Health Insurance. Conversely, the US is the only advanced country in the world without a national health insurance system. Approximately 15% of Americans do not have any health insurance, and America has low healthcare satisfaction as well as poor accessibility, effectiveness, and efficiency [30].

The tendency for poor SRH to have a negative influence on SROH was the same in both countries. SRH had the third-most and first-most negative influence on SROH in Korea and the US, respectively.

With respect to the type of smokers, current smokers showed the highest OR in both Korea and the US, while the OR for poor SROH among current smokers was higher in the US. It is believed that such results may be attributable to the fact that smokers generally have negative SRH [8,9] and that cigarettes are smoked through the mouth, which would also influence SROH. A previous study also reported that current smokers had the poorest SROH, and the effect size was larger in the US than in Korea; thus, the difference in the smoking rate could be a factor that explains the difference in SRH between Korea and the US [9]. However, according to OECD Statistics, the daily smoking rate among the population aged 15 years or older in 2018 was higher in Korea (17.5%) than in the US (10.3%) [10]. Korea having a higher daily smoking rate but the US having a higher OR for poor SROH may be attributable to sociocultural differences when smokers self-rated their own oral health.

Among the indicators of objective oral health, the DT index showed the highest OR for SROH, which was similar to previous studies reporting that the DT and DMFT indices were lower in individuals with better SROH [14,31].

Furthermore, unlike the case with the US, the DT index was included in the top five predictors of SROH in Korea, which suggests that objective oral health status has a greater impact on SROH in Korea than in the US. Thus, oral health projects that first address objective oral health are needed in order to improve SROH of Korean adults, and such projects would ultimately improve oral health-related quality of life [15].

### Strengths and Limitations

This study is significant in that it attempted to identify the differences in SROH between countries using common variables from nationally representative statistical data. However, this study has the following limitations. First, the survey years of the KNHANES and NHANES data were different. In the US, the latest data needed for the study that were published were from 2017–2018. In Korea, the KNHANES Ⅶ (2016–2018) was able to match the US data. However, it was not possible to accurately match the year of investigation of the two countries’ data because the data were provided in a combination of three years. Second, some variables were omitted during the process of extracting common variables investigated in both countries. Periodontal disease, which is one of the top two oral diseases along with dental caries, was omitted because it was not updated in the 2017–2018 NHANES data, while other variables, such as sealants and oral health practice behavior, were also omitted because of the difference in the age of survey participants between the two countries. Third, direct statistical significance could not be confirmed, since data from both countries were not combined for analysis. Finally, there was a methodological bias. In the case of the DMFT index presented as an objective measure of oral health status, it is a factor that is sensitively affected by age; therefore, since this study targeted adults, representing all age groups over 20 years old without age-specific subgroups is a limitation.

Despite these limitations, this study was significant in that it used KNHANES and NHANES data, which are nationally representative statistical data from Korea and the US, respectively, to identify the differences in influencing factors of SROH between the two countries.

## 5. Conclusions

The findings in this study demonstrated that Korean adults had better objective oral health despite more negative ratings of their oral health compared to their US counterparts. The analysis also revealed that the age and type of smokers in Korea, and SRH and household income in the US, had the biggest influence on poor SROH. The study confirmed that these factors could be associated with poorer SROH among Korean adults than among American adults. Therefore, government-led projects and policy-based changes that can improve objective oral health status are needed to enhance SROH in Korea. In particular, SROH tends to evaluate negatively as age increases; therefore, it is necessary to strengthen the coverage of non-reimbursement to increase dental access for the elderly, as well as provide prevention and treatment through the oral health program throughout lifecycle. And future studies are needed to supplement various objective oral health indicators (e.g., periodontal disease) and differences in sociocultural backgrounds between countries.

## Figures and Tables

**Table 1:** General characteristics of the study populations.

	Total	South Korea *n* (Weighted %)		*p*-Value ^b^	Total	US *n* (Weighted %)		*p*-Value ^b^
		Good	Poor			Good	Poor	
Total ^a^	13,065	7935 (61.2)	5130 (38.8)		5562	3675 (72.3)	1887 (27.7)	
Gender				0.023 *				0.063
Male	5724 (42.4)	3395 (59.9)	2329 (40.1)		2697 (48.1)	1738 (70.2)	959 (29.8)	
Female	7341 (57.6)	4540 (62.2)	2801 (37.8)		2865 (51.9)	1937 (74.3)	928 (25.7)	
Age				<0.001 ***				0.099
20–29	1431 (11.5)	1128 (78.0)	303 (22.0)		827 (18.4)	598 (75.3)	229 (24.7)	
30–39	2102 (15.6)	1465 (70.9)	637 (29.1)		859 (17.7)	567 (71.3)	292 (28.7)	
40–49	2418 (17.9)	1625 (68.1)	793 (31.9)		813 (16.2)	505 (71.6)	308 (28.4)	
50–59	2493 (20.1)	1440 (58.9)	1053 (41.1)		919 (18.5)	582 (69.3)	337 (30.7)	
60–69	2254 (17.3)	1153 (51.5)	1101 (48.5)		1103 (15.4)	668 (69.8)	435 (30.2)	
≥70	2367 (17.6)	1124 (47.0)	1243 (53.0)		1041 (13.7)	755 (77.5)	286 (22.5)	
Household income				<0.001 ***				<0.001 ***
Low	2620 (19.7)	1246 (47.4)	1374 (52.6)		906 (13.0)	439 (50.2)	467 (49.8)	
Middle-Low	3173 (24.1)	1789 (56.2)	1384 (43.8)		1324 (20.3)	756 (59.5)	568 (40.5)	
Middle-High	3560 (27.3)	2272 (64.3)	1288 (35.7)		797 (15.3)	530 (69.0)	267 (31.0)	
High	3674 (29.0)	2601 (71.8)	1073 (28.2)		1747 (51.4)	1439 (85.3)	308 (14.7)	
Economic activity				<0.001 ***				0.006 **
Yes	7582 (59.6)	2807 (58.1)	2052 (41.9)		3053 (63.3)	2111 (74.8)	942 (25.2)	
No	4859 (40.4)	4784 (63.7)	2798 (36.3)		2507 (36.7)	1563 (68.0)	944 (32.0)	
Education level				<0.001 ***				<0.001 ***
≤Middle school	3857 (30.4)	1834 (47.4)	2023 (52.6)		1116 (11.3)	555 (49.3)	561 (50.7)	
High school	3903 (32.1)	2394 (61.8)	1509 (38.2)		1323 (27.2)	798 (62.9)	525 (37.1)	
≥University or college	4676 (37.5)	3362 (72.6)	1314 (27.4)		3110 (61.5)	2312 (80.7)	798 (19.3)	
Insurance type				<0.001 ***				<0.001 ***
Employment & county /Private & Medicare	12,452 (95.7)	7661 (61.9)	4791 (38.1)		3605 (74.3)	2610 (78.7)	995 (21.3)	
Government issued /Medicaid	513 (3.5)	221 (43.6)	292 (56.4)		666 (10.7)	348 (55.7)	318 (44.3)	
None	99 (0.8)	52 (57.4)	47 (42.6)		840(15.0)	418 (53.2)	422 (46.8)	
Chronic disease				<0.001 ***				0.003 **
Yes	5889 (44.9)	3151 (53.9)	2738 (46.1)		3840 (65.4)	2476 (70.1)	1364 (29.9)	
No	7173 (55.1)	4782 (67.2)	2391 (32.8)		1722 (34.6)	1199 (76.5)	523 (23.5)	
Type of smokers				<0.001 ***				<0.001 ***
Current smoker	2337 (17.6)	1194 (51.2)	1143 (48.8)		1005 (17.4)	464 (49.8)	541 (50.2)	
Former smoker	2771 (20.8)	1653 (60.4)	1118 (39.6)		1322 (24.8)	853 (71.4)	469 (28.6)	
Never smoked	7819 (61.6)	5014 (64.5)	2805 (35.5)		3235 (57.8)	2358 (79.5)	877 (20.5)	
SRH				<0.001 ***				<0.001 ***
Good	9999 (80.2)	6539 (65.5)	3460 (34.5)		4134 (81.2)	3072 (78.9)	1062 (21.1)	
Poor	2523 (19.8)	1095 (44.7)	1428 (55.3)		1421 (18.8)	598 (44.2)	823 (55.8)	

* *p* < 0.05, ** *p* < 0.01, *** *p* < 0.001. ^a^ by complex sample frequency analysis. ^b^ by complex sample cross-analysis.

**Table 2:** Differences in SROH according to oral health factors.

	Total	South Korea (Mean ± S.E)		*p*-Value	Total	US (Mean ± S.E)		*p*-Value
		Good	Poor			Good	Poor	
Number of present teeth	13,065	24.89 ± 0.12	21.84 ± 0.18	<0.001 ***	5050	24.18 ± 0.25	21.09 ± 0.31	<0.001 ***
DMFT index	13,065	6.50 ± 0.08	8.14 ± 0.16	<0.001 ***	5562	8.98 ± 0.30	10.86 ± 0.31	<0.001 ***
DT index	13,065	0.26 ± 0.01	0.75 ± 0.03	<0.001 ***	5562	0.17 ± 0.02	1.16 ± 0.09	<0.001 ***
MT index	13,065	1.54 ± 0.07	3.26 ± 0.15	<0.001 ***	5562	2.78 ± 0.22	4.95 ± 0.27	<0.001 ***
FT index	13,065	4.70 ± 0.06	4.12 ± 0.09	<0.001 ***	5562	6.04 ± 0.21	4.75 ± 0.28	<0.001 ***

*** *p* < 0.001 by Complex sample descriptive analysis.

**Table 3:** Factors influencing the SROH.

SROH Ref. “Good”	South Korea					US				
	B	S.E.	OR	Exp(B) (95% C.I.)	*p*-Value	B	S.E.	OR	Exp(B) (95% C.I.)	*p*-Value
Gender										
Male	−0.10	0.07	0.90	(0.79–1.02)	0.111	0.20	0.13	1.22	(0.92–1.62)	0.154
Female			1.00	(reference)				1.00	(reference)	
Age										
20–29			1.00	(reference)				1.00	(reference)	
30–39	0.36	0.11	1.43	(1.16–1.77)	<0.001 ***	0.38	0.18	1.46	(0.99–2.15)	0.057
40–49	0.54	0.10	1.72	(1.41–2.09)	<0.001 ***	0.31	0.24	1.36	(0.81–2.29)	0.219
50–59	0.87	0.10	2.39	(1.95–2.91)	<0.001 ***	0.55	0.25	1.72	(1.02–2.92)	0.044 *
60–69	0.89	0.12	2.43	(1.92–3.07)	<0.001 ***	0.36	0.24	1.44	(0.86–2.42)	0.157
≥70	0.70	0.13	2.02	(1.57–2.59)	<0.001 ***	0.22	0.21	1.24	(0.80–1.93)	0.315
Household income										
Low	0.40	0.08	1.49	(1.26–1.75)	<0.001 ***	0.93	0.17	2.54	(1.76–3.67)	<0.001 ***
Middle-Low	0.37	0.07	1.45	(1.27–1.65)	<0.001 ***	0.88	0.14	2.42	(1.80–3.26)	<0.001 ***
Middle-High	0.24	0.06	1.27	(1.13–1.44)	<0.001 ***	0.62	0.13	1.86	(1.41–2.45)	<0.001 ***
High			1.00	(reference)				1.00	(reference)	
Economic activity										
Yes	0.01	0.06	1.01	(0.91–1.13)	0.816	0.28	0.16	1.32	(0.95–1.84)	0.096
No			1.00	(reference)				1.00	(reference)	
Education level										
≤Middle school	0.31	0.08	1.36	(1.17–1.59)	<0.001 ***	0.53	0.12	1.70	(1.32–2.18)	<0.001 ***
High school	0.25	0.06	1.28	(1.13–1.44)	<0.001 ***	0.30	0.14	1.35	(0.99–1.84)	0.055
≥University or college			1.00	(reference)				1.00	(reference)	
Insurance type										
Employment & county /Private & Medicare			1.00	(reference)				1.00	(reference)	
Government issued /Medicaid	−0.05	0.15	0.95	(0.71–1.27)	0.739	0.36	0.24	1.43	(0.87–2.35)	0.150
None	−0.35	0.41	0.71	(0.32–1.59)	0.402	0.47	0.13	1.60	(1.20–2.12)	0.003 **
Chronic disease										
Yes	−0.01	0.06	0.99	(0.89–1.11)	0.862	0.26	0.14	1.30	(0.97–1.74)	0.077
No			1.00	(reference)				1.00	(reference)	
Type of smokers										
Current smoker	0.61	0.08	1.85	(1.58–2.16)	<0.001 ***	0.68	0.10	1.97	(1.59–2.44)	<0.001 ***
Former smoker	0.22	0.06	1.24	(1.08–1.44)	0.003 **	0.26	0.11	1.29	(1.03–1.63)	0.032 *
Never smoked			1.00	(reference)				1.00	(reference)	
SRH										
Good			1.00	(reference)				1.00	(reference)	
Poor	0.58	0.06	1.79	(1.59–2.02)	<0.001 ***	1.20	0.14	3.32	(2.46–4.48)	<0.001 ***
Number of Present teeth	−0.06	0.00	0.97	(0.96–0.98)	<0.001 ***	−0.05	0.01	0.95	(0.91–1.00)	0.063
DT index	0.38	0.03	1.47	(1.39–1.56)	<0.001 ***	0.62	0.06	1.69	(1.51–1.91)	<0.001 ***
MT index	0.08	0.01	1.02	(1.00–1.04)	0.048 *	0.04	0.01	0.97	(0.93–1.02)	0.170
FT index	−0.04	0.01	1.03	(1.02–1.04)	<0.001 ***	−0.05	0.01	1.03	(1.00–1.06)	0.091

* *p* < 0.05, ** *p* < 0.01, *** *p* < 0.001 by Complex sample Logistic regression analysis. (OR, odds ratio; CI, 95% confidence intervals.

## Data Availability

Publicly available datasets were analyzed in this study. This data can be found here: https://knhanes.kdca.go.kr/knhanes/main.do (accessed on 5 March 2022) (South Korea), https://www.cdc.gov/nchs/nhanes/index.htm (accessed on 5 March 2022) (US).
